# Supplementary material for: Detection of Four Porcine Enteric Coronaviruses Using CRISPR-Cas12a Combined with Multiplex Reverse Transcriptase Loop-Mediated Isothermal Amplification Assay
Source: Viruses. 2022 Apr 17;14(4):833. doi: 10.3390/v14040833 (PMC9032155; doi:10.3390/v14040833)
Supplement: Supplementary file 1 [file viruses-14-00833-s001.zip › viruses-1638079-supplementary.pdf]

Supplementary Information

Detection of four porcine enteric coronaviruses using CRISPR-Cas12a combined with multiplex reverse transcriptase loop-mediated isothermal amplification assay

Jiajia Liu[1#], Dagang Tao[2#], Xinquan Chen[1], Linyuan Shen[1], Li Zhu[1], Bingrong Xu[2], Hailong Liu[2], Shuhong Zhao[2,3,4], Xinyun Li[2,3], Xiangdong Liu[2,3], Shengsong Xie[2,3*], Lili Niu[1*]

[1]College of Animal Science and Technology, Sichuan Agricultural University, Chengdu 611130, China; jjliu0419@foxmail.com (J.L.); chenxinquan0705@foxmail.com (X.C.); shenlinyuan0815@163.com (L.S.); zhuli@sicau.edu.cn (L.Z.)

[2]Key Laboratory of Agricultural Animal Genetics, Breeding and Reproduction of Ministry of Education and Key Lab of Swine Genetics and Breeding of Ministry of Agriculture and Rural Affairs, Huazhong Agricultural University, Wuhan 430070, China; dagangtao@foxmail.com (D.T.); xubingrongxu@163.com (B.X.); hailongliu@webmail.hzau.edu.cn (H.L.); shzhao@mail.hzau.edu.cn (S.Z.); xyli@mail.hzau.edu.cn (X.L.); liuxiangdong@mail.hzau.edu.cn (X.L.)

[3]The Cooperative Innovation Center for Sustainable Pig Production, Huazhong Agricultural University, Wuhan 430070, China

[4]Hubei Hongshan Laboratory, Frontiers Science Center for Animal Breeding and Sustainable Production, Wuhan 430070, China

†These authors contributed equally to the study.

*Correspondences: ssxie@mail.hzau.edu.cn (S.X.); niulili@sicau.edu.cn (L.N.)

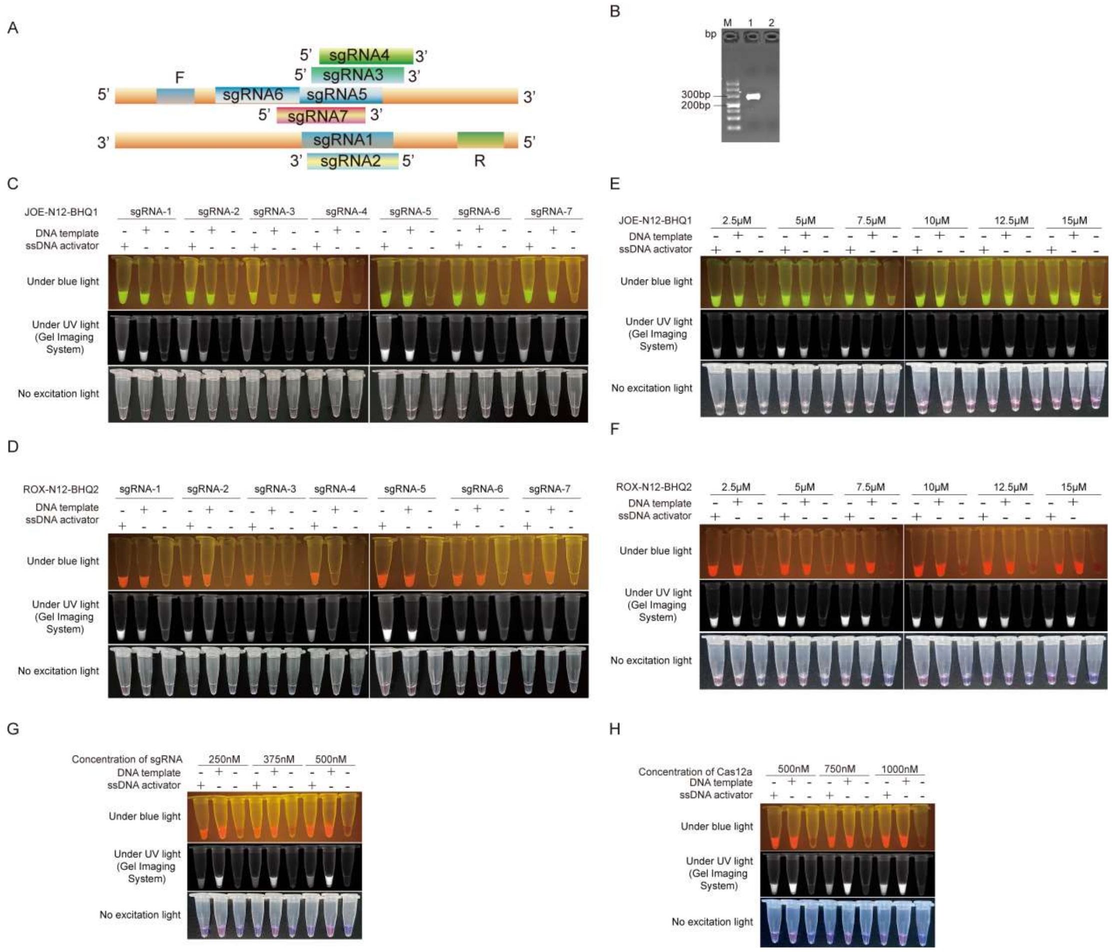

Fig S1. Screening of highly activity sgRNAs for detection of PEDV *ORF3* gene and optimum reaction conditions. (A) Schematic diagram of the PCR primers (F/R) and sgRNAs for detection of *ORF3* gene; **(B)** The *ORF3* amplicon was examined by agarose gel electrophoresis (265 bp); Lane M, DNA Ladder; bp, base pairs; Lane 1, ORF3-plasmid (PUC57-ORF3); Lane 2, non-template control (NTC). **(C)** Colorimetric/fluorescence Cas12a-based assay for detection of PEDV ORF3, JOE-dye ssDNA-FQ reporter was used; **(D)** Colorimetric/fluorescence Cas12a-based assay for detection of PEDV ORF3, ROX-dye ssDNA-FQ reporter was used; **(E)** Evaluation the optimum concentration of JOE-dye ssDNA-FQ reporter for CRISPR/Cas12a-based cleavage assay; **(F)** Evaluation the optimum concentration of ROX-dye ssDNA-FQ reporter for CRISPR/Cas12a-based cleavage assay; **(G)** Evaluation the optimum concentration of sgRNA for CRISPR/Cas12a-based cleavage assay; **(H)** Evaluation the optimum concentration of Cas12a protein for CRISPR/Cas12a-based cleavage assay.

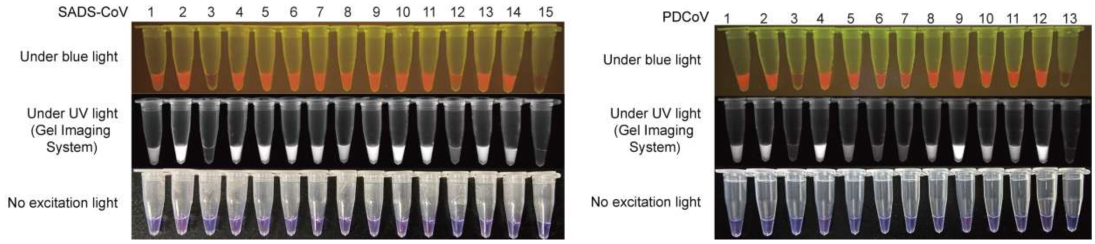

Fig S2. Screening of highly activity sgRNAs for detection of PDCoV *N* and SADS-Co*V N* gene. (A) Colorimetric/fluorescence CRISPR/Cas12a-based assay for detection of SADS-CoV *N* gene; 1-14 represents SADS-CoV *N* gene plasmid used as a template for testing the activity of from sgRNA1 to sgRNA14; 15, non-template control (NTC); **(B)** Colorimetric/fluorescence CRISPR/Cas12a-based assay for detection of PDCoV *N* gene; 1-12 represents PDCoV *N* gene plasmid used as a template for testing the activity of from sgRNA1 to sgRNA12; 13, non-template control (NTC).

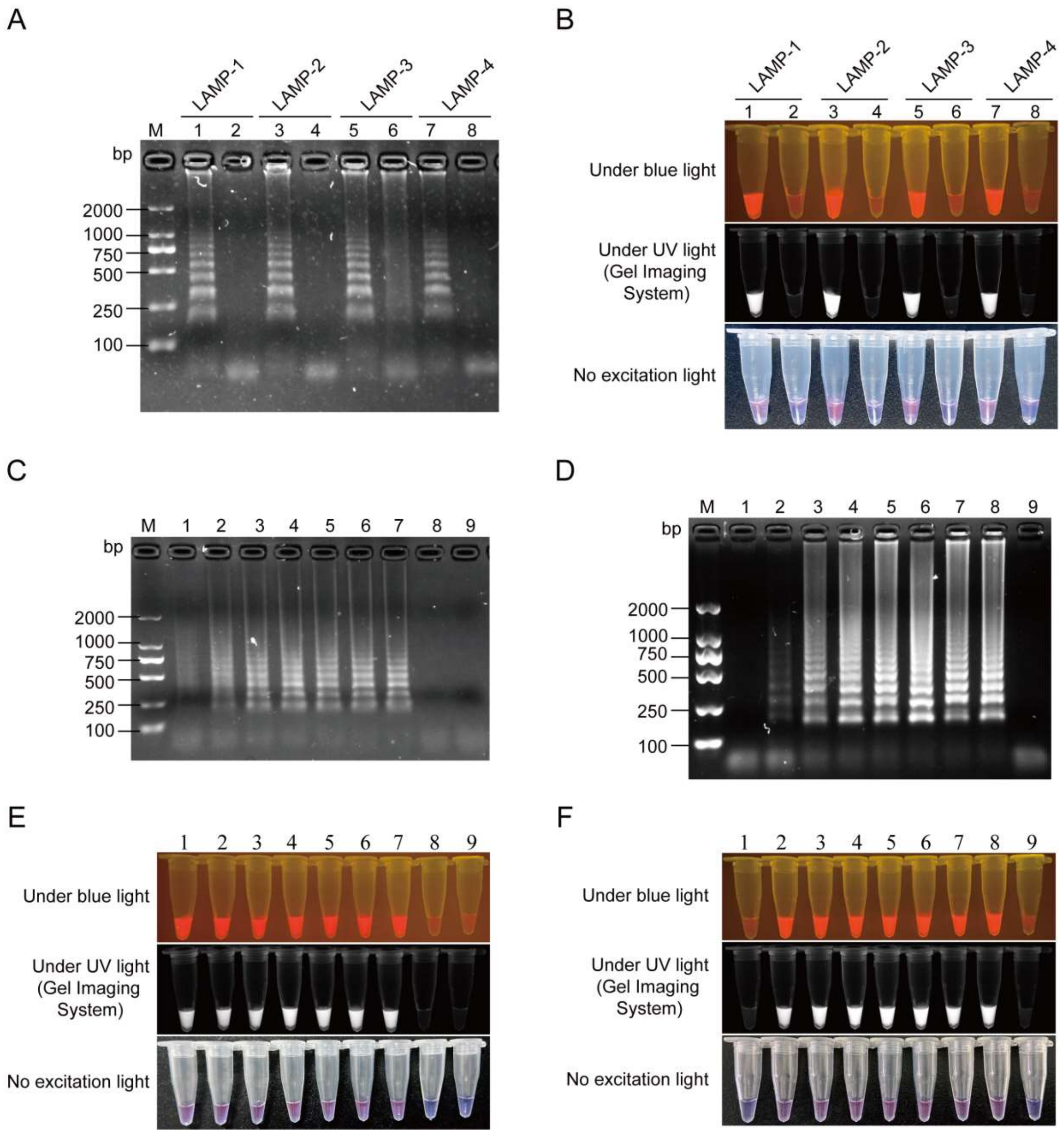

FigS3. Optimization of the reaction conditions in LAMP for PEDV detection. (A) The amplification efficiencies of four LAMP primer pairs by electrophoresis analysis. **(B)** Detecting the fluorescence signal of PEDV *ORF3* gene under the blue (470 nM) and UV lights in CRISPR/Cas12a cleavage assay; Lane M: DNA ladder, bp: base pairs. Lanes or tubes: 1, 3, 5, 7: PEDV-ORF3 plasmid DNA; 2, 4, 6, 8: non-template control. **(C, E)** The effect of the reaction temperature on LAMP-Cas12a assay; Lane M: DNA Ladder 2000 Maker; Lanes or tubes: 1: 53 °C; 2: 55 °C; 3: 57.5 °C; 4: 60 °C; 5: 62.5 °C; 6: 65 °C; 7: 67.5 °C; 8: 71 °C; 9: non-template control (NTC; 65 ºC); **(D, F)** The effect of reaction time on LAMP-Cas12a assay; Lane M: DNA Ladder 2000 Maker; Lanes or tubes: 1: 5 min; Lane 2: 15 min; Lane 3: 20 min; Lane 4: 25 min; Lane 5: 30 min; Lane 6: 35 min; Lane 7: 40 min; Lane 8: 45 min; lane 9: non-template control (NTC; 45 min).

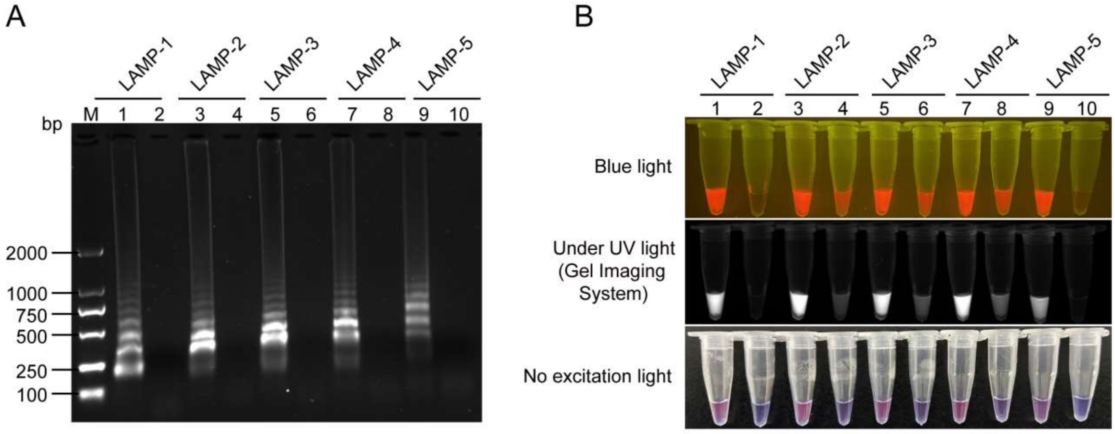

Fig S4. Screening of LAMP primer pairs for detection of TGEV *N* gene. (A) Agarose gel electrophoresis of LAMP products of TGEV *N* gene; **(B)** CRISPR/Cas12a cleavage assay to detection LAMP products of TGEV *N* gene. 1, 3, 5, 7, 9: TGEV-N plasmid DNA; 2, 4, 6, 8, 10: non-template control (NTC).

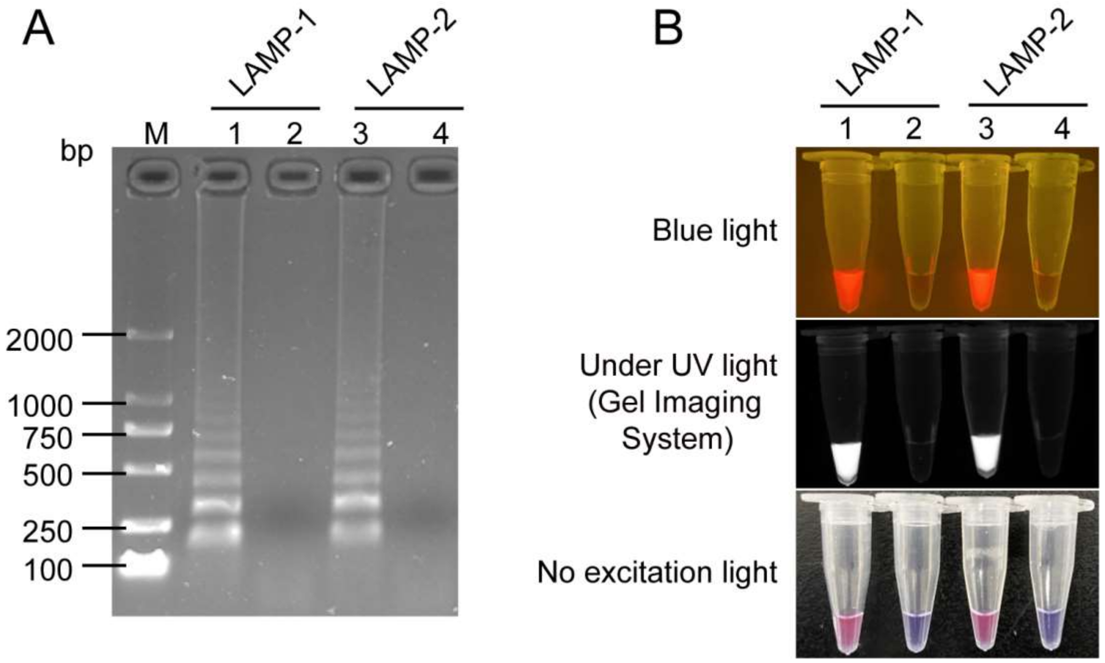

Fig S5. Screening of LAMP primer pairs for detection of PDCoV *N* gene. (A) Agarose gel electrophoresis of LAMP products of PDCoV *N* gene; **(B)** CRISPR/Cas12a cleavage assay to detection LAMP products of PDCoV *N* gene; 1, 3: PDCoV-N plasmid DNA was used as template; 2, 4: non-template control (NTC).

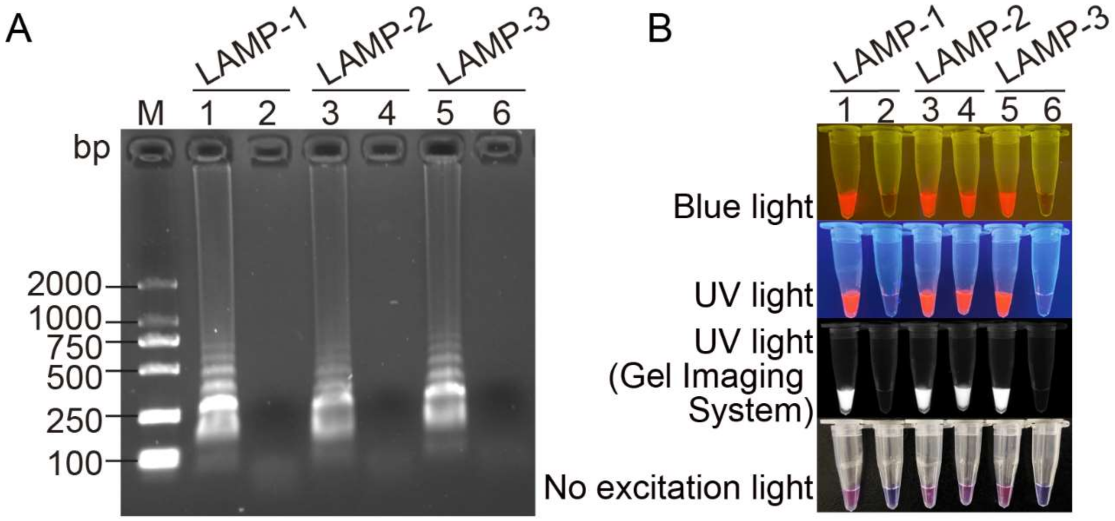

Fig S6. Screening of LAMP primer pairs for detection of SADS-CoV *N* gene. (A) Agarose gel electrophoresis of LAMP products of SADS-CoV *N* gene; **(B)** CRISPR/Cas12a cleavage assay to detection LAMP products of SADS-CoV *N* gene. 1, 3, 5: SADS-CoV-N plasmid DNA was used as template; 2, 4, 6: non-template control (NTC).

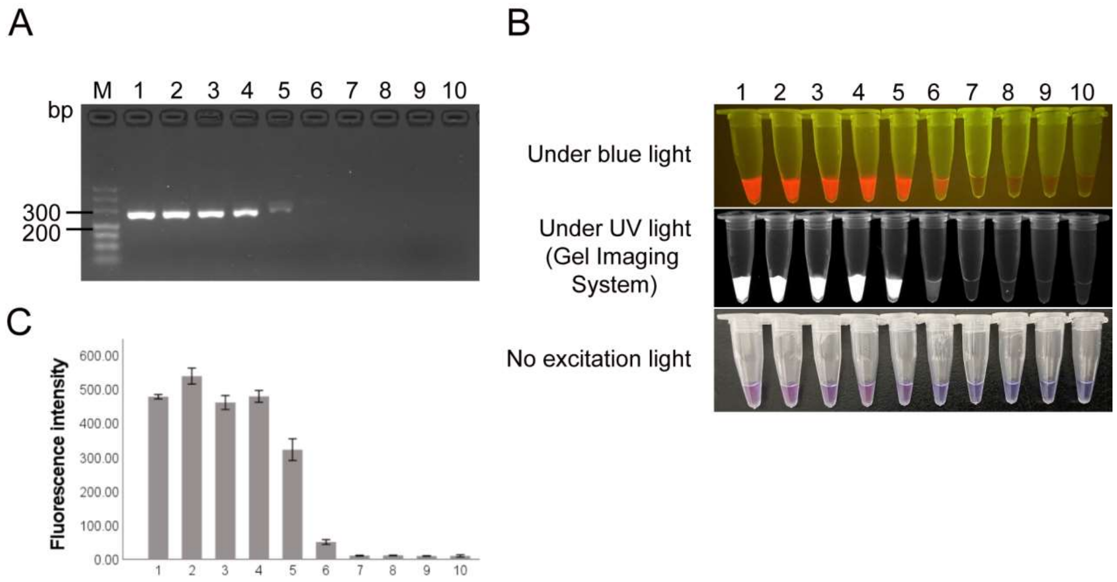

Fig S7. Sensitivity of the PCR-Cas12a assay for detection of *ORF3* gene using serially diluted ORF3-plasmid (8×10^7 to 8×10^0 copies/µL). (A) Products of the PCR reaction by using agarose gel electrophoresis analysis; **(B)** Colorimetric/fluorescence signals from a series of 10-fold dilutions of PUC57-ORF3 plasmid DNA using PCR-Cas12a assay; **(C)** Sensitivity of the PCR-Cas12a assay in a multi-functional microplate reader (n=3); Lane M, DNA Ladder; bp, base pairs; 1, 8×10^7 copies/µL; 2, 8×10^6 copies/µL; 3, 8×10^5 copies/µL; 4, 8×10^4 copies/µL; 5, 8×10^3 copies/µL; 6, 8×10^2 copies/µL; 7, 8×10^1 copies/µL; 8, 8×10^0 copies/µL; 9, 1×10^0 copies/µL; 10, non-template control (NTC).

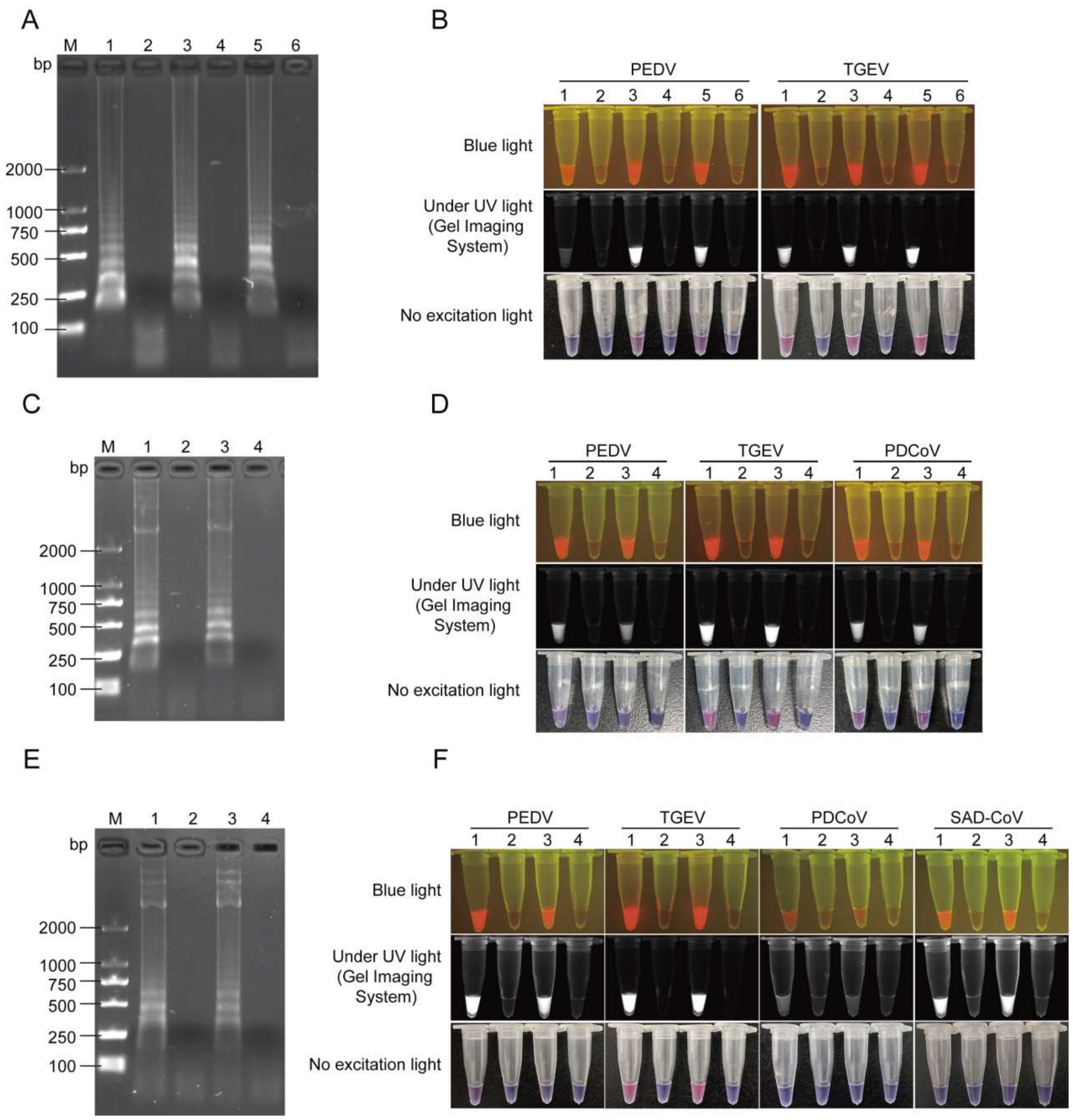

Fig S8. Establishment of multiple LAMP-Cas12a assay for porcine diarrhea coronavirus. (A) The agarose gel electrophoresis for detection of double amplification of PEDV and TGEV; **(B)** The fluorescence signal of Cas12a-based assay for detection of double amplification of PEDV and TGEV; 1, 2: original primer concentration; 3, 4: 1/2 dilution of the original primer concentration; 5, 6: 1/3 dilution of the original primer concentration; 1, 3, 5: plasmid DNA; 2, 4, 6: non-template control (NTC); **(C)** The agarose gel electrophoresis for detection of triple amplification of PEDV, TGEV and PDCoV; **(D)** The fluorescence signal of Cas12a-based assay for detection of triple amplification of PEDV, TGEV and PDCoV; 1, 2: 1/3 dilution of original primer concentration; 3, 4: 1/4 dilution of original primer concentration; **(E)** The agarose gel electrophoresis for detection of quadruple amplification of PEDV, TGEV, PDCoV and SADS-CoV; **(F)** The fluorescence signal of Cas12a-based assay for detection of quadruple

amplification of PEDV, TGEV, PDCoV and SADS-CoV; 1, 2: 1/3 dilution of original primer concentration; 3,4: 1/4 dilution of original primer concentration.

Table S1. Synthetic insert cloned into pUC57 and pMD18T vector.

Oligonucleotides Name	Sequence (5'-3')	Size of oligonucleotides (bp)
**TGEV-N**	AACTTATGTCCGAGAGACTTTGTACCCAAAG<br>GAATAGGTAACAGGGATCAGCAGATTGGTT<br>ATTGGAATAGACAAACTCGCTATCGCATGGT<br>GAAGGGCCAACGTAAAGAGCTTCCTGAAAG<br>GTGGTTCTTCTACTACTTAGGTACTGGACCTC<br>ATGCAGATGCCAAATTTAAAGATAAATTAGA<br>TGGAGTTGTCTGGGTTGCCAAGGATGGTGCC<br>ATGAACAAACCAACCACGCTTGGTAGTCGTG<br>GTGCTAATAATGAATCCAAAGCTTTGAAATT<br>CGATGGTAAAGTGCCAGGCGAATTTCAACTT<br>GAAGTTAACCAGTCAAGGGACAATTCAAGG<br>TCACGCTCTCAATCTAGATCTCGGTCTAGAA<br>ACAGATCTCAATCTAGAGGCAGGCAACAAT<br>CCAATAACAAGAAGGATGACAGTGTAGAAC<br>AAGCTGTTCTTGCCGCACTTAAAAAGTTAGG<br>TGTTGACACAGAAAAACAACAGCAACGTTC<br>TCGTTCTAAATCTAAAGAACGTAGTAACTCT<br>AAAACAAGAGATACTACGCCTAAGAATGAA<br>AACAAACACACCTGGAAGAGAACTGCAGGT<br>AAAGGTGATGTGACAAGATTTTATGGAGCTA<br>GAAGCAGTTCAGCCAATTTTGGTGACAGTGA<br>CCTCGTTGCCAATGGGAGCAGTGCCAAGCAT<br>TACCCACAATTGGCTGAATGTGTTCCATCTGT<br>GTCTAGCATTTTGTTTGGAAGCTATTGGACTT<br>CAAAGGAAGAT	**750**

**pUC57-T7-sgRNA (partial fragment)**	GGATCCCTAATACGACTCACTATAGGGAATT<br>TCTACTGTTGTAGATTGAGACCGAGAGAGGG<br>TCTCATTTTTTAAAGGGCCCGTCGACTGCAG<br>AGGCCTGCATGCAAGCTT	**111**
**PDCoV-N**	GAAAACCATGGCTACTGGCTGCGTTACACCA<br>GACAAAAGCCAGGTGGTACTCCGATTCCTCC<br>ATCCTTTGCCTTTTATTATACTGGCACAGGTC<br>CCAGAGGAAATCTTAAGTATGGTGAACTCCC<br>TCCTAATGATACCCCAGCAACCACTCGTGTT<br>ACTTGGGTTAAGGGTTCGGGAGCTGACACTT<br>CTATTAAACCTCATGTTGCCAAACGCAACCC<br>CAACAATCCTAAACATCAGCTGCTACCTCTC<br>CGATTCCCAACCGGAGATGGCCCAGCTCAA<br>GGTTTCAGAGTTGACCCCTTCAACGCTAGAG<br>GAAGACCTCAGGAGCGTGGAAGTGGCCCAA<br>GATCTCAATCTGTTAACTCCAGAGGCACAGG<br>CAATCAGCCCAGGAAACGCGACCAATCTGC<br>ACCAGCTGCGGTACGTCGTAAGACCCAGCAT<br>CAAGCTCCCAAGCGGACTTTACCCAAGGGTG<br>AAACCATTTCTCAGGTATTTGGCAACCGGTC<br>TCGTACTGGTGCCAATGTCGGCTCTGCAGAC<br>ACTGAGAAGACGGGTATGGCTGATCCTCGCA<br>TCATGGCTCTAGCCGGACATGTGCCTGGTGT<br>TCAGGAAATGTTTTTCGCTGGCCACCTTGAG<br>AGCAACTTTCAGGCGGGGGCAATTACCCTTA<br>CCTTCTCCTACTCAATCACAGTCAAGGAGGG<br>TTTTCCTGACTATGGGAGACTTAAGGATGCG<br>CTCAATACGGTCGTTAACCAGACCTATGAGC<br>CACCTACCAAACCAACTAAGGACAAGAAGC	**900**

	CTGACAAACAAGTCCAGTCTGCTAAACCCA<br>AACAGCAGAAGAAACCTAAAAAGGTAACTC<br>TGCCAGCAGACAAACAGGATTGGGAGTGGG<br>ATGATGCTTTTGAGATAAAGCAGGAATCAGC<br>AGCGTAG	
**SAD-CoV-N**	CCAACGTAAAGATCAGCCTTCTAACTGGCACT<br>TTTATTACCTTGGTACTGGTCCTCACGCAGAT<br>GCTCCTTTCAGGAAACGGATTCAGGGTGTGCA<br>TTGGGTCGCTGTTGACGGTGCTAAAACTAGCC<br>CCACAGGTCTTGGTGTTCGCAATCGTAACAAA<br>GAACCTGCTACACCTCAGTTTGGGTTTCAATT<br>ACCACCAGACCTGACTGTTGTTGAGGTTACTT<br>CTAGAAGTGCTTCACGTTCACAGTCTCGTTCT<br>CGCAATCAAAGTCAAAGCCGCAGTGGTGCTC<br>AGACACCTCGTGCTCAACAGCCGTCACAGTCT<br>GTTGACATTGTTGCTGCAGTTAAACAAGCTTT<br>GGCAGACTTGGGCATAGCTTCTAGCCAGTCCA<br>GGCCTCAAAGTGGTAAAAATACACCCAAACC<br>AAGAAGCAGAGCTGTCTCACCTGCACCTGCC<br>CCTAAACCGGCTCGTAAGCAGATGGACAAAC<br>CTGAATGGAAGCGTGTTCCTAATTCTGAGGAG<br>GACGTGCGTAAATGCTTTGGTCCTCGCTCAGT<br>TTCTAGAAATTTTGGTGACAGTGACCTCGTTC<br>AGCACGGTGTTGAAGCTAAGCACTTTCCAAC<br>AATTGCTGAGTTGCTTCCGACACAAGCTGCAC<br>TAGCCTTTGGTAGTGAAATCACAACCAAAGA<br>GTCTGGTGAATTTGTAGAAGTCACCTATCACT<br>ATGTAATGAAGGTCCCCAAGACTGATAAAAA<br>TCTACCCAGATTTCTTGAGCA	**750**

**PEDV-ORF3**	AGACAAGCTTCAAATGTGACGGGTTTTCTTTT<br>CACCAGTGTTTTTATCTACTTCTTTGCACTGTT<br>TAAAGCGTCTTCTTTGAGGCGCAATTATATTA<br>TGTTGGCAGCGCGTTTTGCTGTCATTGTTCTTT<br>ATTGCCCACTTTTATATTATTGTGGTGCATTTT<br>TAGATGCAACTATTATTTGTTGCACACTTATTG<br>GCAGGCTTTGTTTAGTCTGCTTTTACTCCTGGC<br>GCTATAAAAATGCGCTCTTTATTATCTTTAATA<br>CTACGACACTTTCTTTCCTCAATGGTAAAGCA<br>GCTTATTATGACGGCAAATCCATTGT	**320**

Table S2. Primers and oligos used in this study.

**Application**	**Oligonucleotides Name**	**Primer sequence (5'-3')**	**Target ID**	**Production Length(bp)**
**PCR primers**	SADS-N-T7-F	TAATACGACTCACTATAGGCAGGAAACG<br>GATTCAGGG	MG775253.1	**478**
	SADS-N-R	GGACCAAAGCATTTACGC		
	PEDV-ORF3-F	GAGGAAAGAAAGTGTCGTAG	KJ642645.1	**265**
	PEDV-ORF3-R	GACGGGTTTTCTTTTCAC		
**sgRNA (For In Vitro synthesis)**	T7-crRNA-F	TCGCTATTACGCCAGCTGGC		
	PEDV-sgR1-R	AGTCTGCTTTTACTCCTGGCGCTAATCTAC<br>AACAGTAGAAAT	PEDV-sgRNA1	
	PEDV-sgR2-R	TCTGCTTTTACTCCTGGCGCTATAATCTAC<br>AACAGTAGAAAT	PEDV-sgRNA2	
	PEDV-sgR3-R	GCGCATTTTTATAGCGCCAGGAGTATCTAC<br>AACAGTAGAAAT	PEDV-sgRNA3	
	PEDV-sgR4-R	AGCGCATTTTTATAGCGCCAGGAGATCTA<br>CAACAGTAGAAAT	PEDV-sgRNA4	
	PEDV-sgR5-R	ATAGCGCCAGGAGTAAAAGCAGACATCT<br>ACAACAGTAGAAAT	PEDV-sgRNA5	
	PEDV-sgR6-R	CAAAGCCTGCCAATAAGTGTGCAAATCTA<br>CAACAGTAGAAAT	PEDV-sgRNA6	

	PEDV-sgR7-R	CGCCAGGAGTAAAAGCAGACTAAAATCTACAACAGTAGAAAT	PEDV-sgRNA7
	TGEV-N-sgR1-R	GCTCTCAATCTAGATCTCGGTCTAATCTACAACAGTAGAAAT	TGEV-sgRNA1
	TGEV-N-sgR2-R	AGAACAAGCTGTTCTTGCCGCACTATCTACAACAGTAGAAAT	TGEV-sgRNA2
	TGEV-N-sgR3-R	AAAACAACAGCAACGTTCTCGTTCATCTACAACAGTAGAAAT	TGEV-sgRNA3
	TGEV-N-sgR4-R	TAAATCTAAAGAACGTAGTAACTCATCTACAACAGTAGAAAT	TGEV-sgRNA4
	TGEV-N-sgR5-R	CTTAAAAAGTTAGGTGTTGACACAATCTACAACAGTAGAAAT	TGEV-sgRNA5
	TGEV-N-sgR6-R	CCCTTGACTGGTTAACTTCAAGTTATCTACAACAGTAGAAAT	TGEV-sgRNA6
	SADS-sg1-R	GTCTGTTGACATTGTTGCTGCAGTATCTACAACAGTAGAAAT	SADSCoV-sgRNA1
	SADS-sg2-R	AGCTGTCTCACCTGCACCTGCCCCATCTACAACAGTAGAAAT	SADSCoV-sgRNA2
**sgRNA (For In Vitro synthesis)**	SADS-sg3-R	CGTGCTGAACGAGGTCACTGTCACATCTACAACAGTAGAAAT	SADSCoV-sgRNA3
	SADS-sg4-R	TCCTAATTCTGAGGAGGACGTGCGATCTACAACAGTAGAAAT	SADSCoV-sgRNA4
	SADS-sg5-R	GCTAGAAGCTATGCCCAAGTCTGCATCTACAACAGTAGAAAT	SADSCoV-sgRNA5
	SADS-sg6-R	TAGCCAGTCCAGGCCTCAAAGTGGATCTACAACAGTAGAAAT	SADSCoV-sgRNA6
	SADS-sg7-R	ATAGCTTCTAGCCAGTCCAGGCCTATCTACAACAGTAGAAAT	SADSCoV-sgRNA7
	SADS-sg8-R	GCTTTGGTCCTCGCTCAGTTTCTAATCTACAACAGTAGAAAT	SADSCoV-sgRNA8
	SADS-sg9-R	AATTTCTAGAAACTGAGCGAGGACATCTACAACAGTAGAAAT	SADSCoV-sgRNA9
	SADS-sg10-R	TAAACCGGCTCGTAAGCAGATGGAATCTACAACAGTAGAAAT	SADSCoV-sgRNA10
	SADS-sg11-R	TCAGGTCTGGTGGTAATTGAAACCATCTACAACAGTAGAAAT	SADSCoV-sgRNA11
	SADS-sg12-R	CAACAGTCAGGTCTGGTGGTAATTATCTACAACAGTAGAAAT	SADSCoV-sgRNA12

	SADS-sg13-R	CGTTCACAGTCTCGTTCTCGCAATATCTAC AACAGTAGAAAT	SADSCoV-sgRNA13
	SADS-sg14-R	CAGTCTCGTTCTCGCAATCAAAGTATCTA CAACAGTAGAAAT	SADSCoV-sgRNA14
	PDCoV-sg1-R	TCTAGCGTTGAAGGGGTCAACTCTATCTA CAACAGTAGAAAT	PDCoV-sgRNA1
	PDCoV-sg2-R	GCCGGACATGTGCCTGGTGTTCAGATCTA CAACAGTAGAAAT	PDCoV-sgRNA2
**sgRNA (For In Vitro synthesis)**	PDCoV-sg3-R	CTGAGAAATGGTTTCACCCTTGGGATCTA CAACAGTAGAAAT	PDCoV-sgRNA3
	PDCoV-sg4-R	CAGAGGCACAGGCAATCAGCCCAGATCT ACAACAGTAGAAAT	PDCoV-sgRNA4
	PDCoV-sg5-R	CACTTCTATTAAACCTCATGTTGCATCTAC AACAGTAGAAAT	PDCoV-sgRNA5
	PDCoV-sg6-R	ATCCTTAAGTCTCCCATAGTCAGGATCTAC AACAGTAGAAAT	PDCoV-sgRNA6
	PDCoV-sg7-R	CATCCTTAAGTCTCCCATAGTCAGATCTAC AACAGTAGAAAT	PDCoV-sgRNA7
	PDCoV-sg8-R	GGGTTCGGGAGCTGACACTTCTATATCTA CAACAGTAGAAAT	PDCoV-sgRNA8
	PDCoV-sg9-R	TGGCACCAGTACGAGACCGGTTGCATCTA CAACAGTAGAAAT	PDCoV-sgRNA9
	PDCoV-sg10-R	CGAGACCGGTTGCCAAATACCTGAATCTA CAACAGTAGAAAT	PDCoV-sgRNA10
	PDCoV-sg11-R	AGTTGCTCTCAAGGTGGCCAGCGAATCTA CAACAGTAGAAAT	PDCoV-sgRNA11
	PDCoV-sg12-R	AAGTTGCTCTCAAGGTGGCCAGCGATCTA CAACAGTAGAAAT	PDCoV-sgRNA12
**LAMP primers**	PEDV-L1-F3	GCACTGTTTAAAGCGTCTT	
	PEDV-L1-B3	AGGAAAGAAAGTGTCGTAGT	
	PEDV-L1-FIP	GCACCACAATAATATAAAGTGGGCGGC GCAATTATATTATGTTGG	
	PEDV-L1-BIP	TTGTTGCACACTTATTGGCAGAAGAGCGC ATTTTTATAGCG	
	PEDV-L1-LF	AAGAACAATGACAGCAAAACGC	
	PEDV-L1-LB	TGTTTAGTCTGCTTTTACTCCTGG	
	PEDV-L2-F3	GCACTGTTTAAAGCGTCTT	

	PEDV-L2-B3	GAGGAAAGAAAGTGTCGTAGT
	PEDV-L2-FIP	GCACCACAATAATATAAAAGTGGGCGGC GCAATTATATTATGTTGG
	PEDV-L2-BIP	TTGTTGCACACTTATTGGCAGAAGAGCGC ATTTTTATAGCG
	PEDV-L2-LF	AAGAACAATGACAGCAAAACGC
	PEDV-L2-LB	TGTTTAGTCTGCTTTTACTCCTGG
	PEDV-L3-F3	GTTTAAAGCGTCTTCTTTGAG
	PEDV-L3-B3	AGGAAAGAAAGTGTCGTAGT
**LAMP primers**	PEDV-L3-FIP	GCACCACAATAATATAAAAGTGGGCGCG CAATTATATTATGTTGGC
	PEDV-L3-BIP	TTGTTGCACACTTATTGGCAGAGAGCGCA TTTTTATAGCG
	PEDV-L3-LF	AAGAACAATGACAGCAAAACGC
	PEDV-L3-LB	TGTTTAGTCTGCTTTTACTCCTGG
	PEDV-L4-F3	GTTTAAAGCGTCTTCTTTGAG
	PEDV-L4-B3	GAGGAAAGAAAGTGTCGTAGT
	PEDV-L4-FIP	GCACCACAATAATATAAAAGTGGGCGCG CAATTATATTATGTTGGC
	PEDV-L4-BIP	TTGTTGCACACTTATTGGCAGAAGAGCGC ATTTTTATAGCG
	PEDV-L4-LF	AAGAACAATGACAGCAAAACGC
	PEDV-L4-LB	TGTTTAGTCTGCTTTTACTCCTGG
	TGEV-L1-F3	GGCAACAATCCAATAACAAGAA
	TGEV-L1-B3	TGAACTGCTTCTAGCTCCA
	TGEV-L1-FIP	GTTGCTGTTGTTTTTCTGTGTCAGATGACA GTGTAGAACAAGC
	TGEV-L1-BIP	ACTCTAAAACAAGAGATACTACGCCCAC ATCACCTTTACCTGCA
	TGEV-L1-LF	ACCTAACTTTTTAAGTGCGGCAAG
	TGEV-L1-LB	GAATGAAAACAAACACACCTGGAAG
	TGEV-L2-F3	AATTTCAACTTGAAGTTAACCAG
	TGEV-L2-B3	AACGAGAACGTTGCTGTT

	TGEV-L2-FIP	TGCCTCTAGATTGAGATCTGTTTCTTCAAGGGACAATTCAAGGT
	TGEV-L2-BIP	AGAAGGATGACAGTGTAGAACAAGCGTTTTTCTGTGTCAACACCTA
	TGEV-L2-LF	CGAGATCTAGATTGAGAGCGTG
	TGEV-L2-LB	GTTCTTGCCGCACTTAA
	TGEV-L3-F3	CCAGGCGAATTTCAACTTG
	TGEV-L3-B3	GAACGAGAACGTTGCTGT
	TGEV-L3-FIP	TGCCTCTAGATTGAGATCTGTTTCTTCAAGGGACAATTCAAGGTC
	TGEV-L3-BIP	AGAAGGATGACAGTGTAGAACAAGCTGTTTTTCTGTGTCAACACC
	TGEV-L3-LF	GACCGAGATCTAGATTGAGAGCG
	TGEV-L3-LB	TGTTCTTGCCGCACTTAAAAAGTT
	TGEV-L4-F3	GGCGAATTTCAACTTGAAGT
	TGEV-L4-B3	GAACGAGAACGTTGCTGT
	TGEV-L4-FIP	GCCTCTAGATTGAGATCTGTTTCTGTCAAGGGACAATTCAAGGT
	TGEV-L4-BIP	GAAGGATGACAGTGTAGAACAAGCTGTTTTTCTGTGTCAACACC
	TGEV-L4-LF	CCGAGATCTAGATTGAGAGCGTG
	TGEV-L4-LB	TGTTCTTGCCGCACTTAAAAAGT
**LAMP primers**	TGEV-L5-F3	TCAAGGTCACGCTCTCAA
	TGEV-L5-B3	GTTTGTTTTCATTCTTAGGCG
	TGEV-L5-FIP	GCTTGTTCTACACTGTCATCCTTCTATCTCGGTCTAGAAACAGATC
	TGEV-L5-BIP	GTTAGGTGTTGACACAGAAAAACAAATCTCTTGTTTTAGAGTTACTACGT
	TGEV-L5-LF	TGTTGCCTGCCTCTAGATTGA
	TGEV-L5-LB	CAGCAACGTTCTCGTTCTAAATCT
	SADSCOV-L1-F3	ACAAGCTTTGGCAGACTTGG
	SADSCOV-L1-B3	GAGCGAGGACCAAAGCATT
	SADSCOV-L1-FIP	TGCAGGTGAGACAGCTCTGCAGCTTCTAGCCAGTCCAGG

	SADSCOV-L1-BIP	GCCCCTAAACCGGCTCGTAAGCGCACGT CCTCCTCAGAA
	SADSCOV-L1-LF	CTTCTTGGTTTGGGTGTAT
	SADSCOV-L1-LB	ACAAACCTGAATGGAAGCGTGT
	SADSCOV-L2-F3	GCTCAGACACCTCGTGCT
	SADSCOV-L2-B3	CCATCTGCTTACGAGCCG
	SADSCOV-L2-FIP	AGCTATGCCCAAGTCTGCCAAACCGTCA CAGTCTGTTGACAT
	SADSCOV-L2-BIP	TCTAGCCAGTCCAGGCCTCAAAGGTGCA GGTGAGACAGCT
	SADSCOV-L2-LF	GCTTGTTTAACTGCAGCAACA
	SADSCOV-L2-LB	AAATACACCCAAACCAAGAAGC
**LAMP primers**	SADSCOV-L3-F3	ACAAGCTTTGGCAGACTTGG
	SADSCOV-L3-B3	AGAAACTGAGCGAGGACCAA
	SADSCOV-L3-FIP	TGCAGGTGAGACAGCTCTGCTTAGCTTCT AGCCAGTCCAGG
	SADSCOV-L3-BIP	TGCCCCTAAACCGGCTCGTAACGCACGTC CTCCTCAGAA
	SADSCOV-L3-LF	TCTTGGTTTGGGTGTAT
	SADSCOV-L3-LB	CAAACCTGAATGGAAGCGTG
	PDCOV-L1-F3	GCAATCAGCCCAGGAAACG
	PDCOV-L1-B3	AACACCAGGCACATGTCC
	PDCOV-L1-FIP	TCACCCTTGGGTAAAGTCCGCCAGCTGCG GTACGTCGTA
	PDCOV-L1-BIP	ATGTCGGCTCTGCAGACACTGGCTAGAGC CATGATGCGAG
	PDCOV-L1-LF	TGGGAGCTTGATGCTGG
	PDCOV-L1-LB	AGACGGGTATGGCTGATC
	PDCOV-L2-F3	GCAATCAGCCCAGGAAACG
	PDCOV-L2-B3	TCCTGAACACCAGGCACAT
	PDCOV-L2-FIP	TCACCCTTGGGTAAAGTCCGCCAGCTGCG GTACGTCGTA
	PDCOV-L2-BIP	ATGTCGGCTCTGCAGACACTGGTCCGGCT AGAGCCATGA

	PDCOV-L2-LF	TGGGAGCTTGATGCTGG
	PDCOV-L2-LB	GGTATGGCTGATCCTCGC
**ssDNA-FQ reporters**	**ROX-N12-BHQ2**	/5′-**ROX**/GTATCCAGTGCG/3′**BHQ2/**
	**JOE-N12-BHQ1**	/5′-J**OE**/GTATCCAGTGCG/3′**BHQ1**/
